# Multi-omic molecular characterization and diagnostic biomarkers for occult hepatitis B infection and HBsAg-positive hepatitis B infection

**DOI:** 10.3389/fendo.2024.1409079

**Published:** 2024-11-12

**Authors:** Xinyi Jiang, Jinyue Tian, Li Song, Jiao Meng, Zhenkun Yang, Weizhen Qiao, Jian Zou

**Affiliations:** Department of Laboratory Medicine, The Affiliated Wuxi People’s Hospital of Nanjing Medical University, Wuxi People’s Hospital, Wuxi Medical Center, Nanjing Medical University, Wuxi, Jiangsu, China

**Keywords:** occult hepatitis B infection, HBsAg-positive hepatitis B infection, biomarkers, multi-omics, proteomics

## Abstract

**Background:**

The pathological and physiological characteristics between HBsAg-positive HBV infection and occult hepatitis B infection (OBI) are currently unclear. This study aimed to explore the immune microenvironment in the peripheral circulation of OBI patients through integration of proteomic and metabolomic sequencing, and to identify molecular biomarkers for clinical diagnosis of HBsAg-positive HBV and OBI.

**Methods:**

This research involved collection of plasma from 20 patients with OBI (negative for HBsAg but positive for HBV DNA, with HBV DNA levels < 200 IU/mL), 20 patients with HBsAg-positive HBV infection, and 10 healthy individuals. Mass spectrometry-based detection was used to analyze the proteome, while nuclear magnetic resonance spectroscopy was employed to study the metabolomic phenotypes. Differential molecule analysis, pathway enrichment and functional annotation, as well as weighted correlation network analysis (WGCNA), were conducted to uncover the characteristics of HBV-related liver disease. Prognostic biomarkers were identified using machine learning algorithms, and their validity was confirmed in a larger cohort using enzyme linked immunosorbent assay (ELISA).

**Results:**

HBsAg-positive HBV individuals showed higher ALT levels (*p*=0.010) when compared to OBI patients. The influence of HBV infection on metabolic functions and inflammation was evident through the analysis of distinct metabolic pathways in HBsAg-positive HBV and OBI groups. Tissue tracing demonstrated a connection between Kupffer cells and HBsAg-positive HBV infection, as well as between hepatocytes and OBI. Immune profiling revealed the correlation between CD4 Tem cells, memory B cells and OBI, enabling a rapid response to infection reactivation through cytokine secretion and antibody production. A machine learning-constructed and significantly expressed molecule-based diagnostic model effectively differentiated HBsAg-positive and OBI groups (AUC values > 0.8). ELISA assay confirmed the elevation of FGB and FGG in OBI samples, suggesting their potential as biomarkers for distinguishing OBI from HBsAg-positive infection.

**Conclusions:**

The immune microenvironment and metabolic status of HBsAg-positive HBV patients and OBI patients vary significantly. The machine learning-based diagnostic model described herein displayed impressive classification accuracy, presenting a non-invasive means of differentiating between OBI and HBsAg-positive HBV infections.

## 1 Introduction

Occult hepatitis B infection (OBI) is a unique infectious status during hepatitis B virus (HBV) infection. It is identified by the existence of low levels of HBV DNA in the liver and/or blood of individuals who yield negative results for hepatitis B surface antigen (HBsAg) with current tests (1).

The prevalence of OBI exhibits distinct regional characteristics. A meta-analysis conducted across multiple databases revealed that the prevalence of OBI among blood donors was 0.06%, 0.12%, and 0.98% in countries with low, intermediate, and high endemicity of HBV, respectively (2). This pattern closely reflected the endemicity of HBV. In the general healthy population (3), the prevalence of OBI was 0.8%, while in the overall population, it was 3.8%. However, in high-risk groups such as HIV-infected individuals and children born to mothers with HBsAg-positive status, the prevalence of OBI was significantly higher, reaching up to 24.2% and 6.4%, respectively.

The prevalence of OBI cannot be ignored, and the risk of OBI is gradually being revealed. HBV infection can be transmitted by OBI patients with only 16 copies/mL of HBV DNA (4), and OBI has been shown to be associated with advanced chronic liver disease (5), especially cirrhosis (6) and hepatocellular carcinoma (HCC) (1, 7). OBI patients were found to have a higher incidence of HCC, as well as more advanced tumor histological grades and earlier age of HCC diagnosis, compared to patients without OBI (7). The persistence of OBI can lead to HBV DNA integration in the hepatocyte genome, and the production of pro-oncogenic proteins, such as HBx protein and mutated surface proteins, which accelerate the development of HBV-related liver diseases (8). Additionally, in OBI patients with a history of HBV infection, immunosuppression or chemotherapy status may restart viral replication and trigger reactivation of infection.

Despite the long-standing description and global recognition of its clinical implications, the molecular mechanisms and immune microenvironment of OBI have not been thoroughly explored. Furthermore, while liver biopsy is the preferred method for verifying OBI, its invasiveness hinders its widespread use. Nucleic acid testing is used to diagnose occult HBV infection by amplifying a specific gene fragment of HBV DNA. However, due to the window period of virus infection and the virus genome’s tendency to mutate, there is a risk of missed detection. It is crucial to investigate OBI through emerging methods and establish new molecular biomarkers that strongly associate with the clinical diagnosis of OBI. As differentiating OBI from HBsAg positive infection may help identify distinct OBI markers for additional diagnostic purposes, better understand the various stages of HBV infection, monitor treatment progress, and allow for the exploration of OBI mechanisms.

As proteomic and metabolomic detection methods for small amounts of clinical samples continue to advance, researchers are increasingly using these techniques to develop prognostic models and identify novel biomarkers. A previous study used plasma from CHB patients and patients with acute-on-chronic liver failure (HBV-ACLF) (9) to screen potential protein molecular markers associated with specific symptoms. Then, they performed targeted quantitative detection of these protein molecules in a validation cohort to evaluate and compare their diagnostic and prognostic performance. This mirrors the classical process of identifying molecular markers of other diseases.

The main goal of this research was to analyze the features of the circulating immune microenvironment in individuals with OBI and HBsAg-positive infection, by merging proteomic and metabolomic outcomes. Another aim was to combine the proteomic and metabolomic results to identify novel molecular markers that could be used for clinical diagnosis.

## 2 Methods

### 2.1 Patient cohort and sample preparation

In the discovery cohorts, a total of 50 samples were collected, including 20 samples with occult HBV infection (OBI), 20 samples from HBsAg positive HBV infection, and 10 samples from healthy controls. In the validation cohorts, a total of 289 samples were collected, including 78 OBI samples, 82 HBsAg-positive and anti-HBc-positive samples, 71 HBsAg-positive, anti-HBe-positive and anti-HBc-positive samples, and 58 HBsAg-positive, HBeAg-positive and anti-HBc-positive samples (**Supplementary Table S1**). These samples were collected from participants who were enrolled at Wuxi Red Cross Blood Center and Wuxi People’s Hospital between November 2017 and December 2023. This study was approved by the Research Ethics Committee of Wuxi People’s Hospital (KY23164).

The blood samples were centrifuged at 3000 rpm for 10 min, plasma was collected and stored at −80°C for the following research. The HBV DNA in the plasma were extracted and quantified using a diagnostic kit for quantification of HBV DNA (IU/mL, PCR-Fluorescence Probing, Shengxiang, China) on LightCycle 480 II detection system according to the manufacturer’s instructions. Samples with viral load below 100 IU/mL were reported as positive but under detectable limit. The HBV biomarker module was validated through a retest of five serological markers (HBsAg, anti-HBs, HBeAg, anti-HBe, and anti-HBc) for HBV infection using the Architect-i2000 chemiluminescence immune analyzer and the relevant reagents from Abbott Laboratories, Abbott Park, IL, USA. The diagnosis of HBV infection and OBI was based on clinical and laboratory results according to two clinical guidelines (1, 10). The diagnostic criteria for OBI include the absence HBsAg in blood as detected by current assays, but the presence of HBV DNA in blood and/or liver tissues.

### 2.2 Proteome and metabolome analysis

Plasma proteins were extracted and then subjected to trypsin digestion according to the manufacturer’s instructions (11). Following that, the samples underwent analysis using LC-MS equipment, which comprised of an EASY-nLC 1200 ultra-high-pressure system (Thermo Fisher Scientific), a Fusion Lumos Orbitrap (Thermo Fisher Scientific), and a nano-electrospray ion source (Thermo Fisher Scientific).

The peptides were dissolved, and loaded onto a trap column at the highest pressure. Peptides were separated on a specific column using a linear gradient at specific flow rate and time. The study utilized data-independent MS analysis (DIA) to investigate the proteome results, with the data being analyzed using Firmiana software for peptide identification and protein quantification (12). Subsequently, the data was compared against the UniProt human protein database. To ensure accuracy, the false discovery rate (FDR) at the PSM level was kept below 1% through the target-decoy strategy, and protein quantification was based on the total intensities of the identified peptides.

Metabolome analysis was conducted using nuclear magnetic resonance (NMR) spectroscopy (13). Plasma samples were heated at 56°C for 30 minutes and then analyzed using a 600 MHz NMR spectrometer with adjustments. The samples were mixed with a phosphate buffer and placed in NMR tube for analysis. A total of 348 quantitative parameters were utilized to describe the metabolomic profiles of each plasma sample, encompassing lipoproteins, glycoproteins, small metabolites, and fatty acids. Further information and specific parameters can be found in previously published articles (11, 14).

### 2.3 Differential molecular analysis and pathway enrichment analysis

The limma package in R was applied to screen for differentially expressed molecules, with absolute fold-change >1.5 and adjusted p < 0.05 considered as cutoff value (15).

The biological characteristics of various groups and the differentially-expressed molecules in HBV patients and healthy controls were identified through pathway enrichment analysis with Reactome using the cluster profiler package in R. The significance of pathway enrichment was assessed using the Fisher exact test, and pathways meeting an FDR threshold of 0.05 were considered significantly regulated.

### 2.4 Weighted gene correlation network analysis

WGCNA was performed in R using a WGCNA package (16). Following the application of the WGCNA algorithm for dimensionality reduction clustering, the scores for each module in every sample were determined. The optimized correlation analysis function “cor” in WGCNA was then utilized to assess the correlation between module scores and clinical characteristics. A correlation coefficient exceeding 0.25 signified the existence of an interaction relationship.

### 2.5 Identification of tissue-enhanced proteins

To better understand the impact of different HBV infection status on patients, plasma proteome data was mapped with the Human Protein Atla (HPA) database and determined different expressions of tissue-enhanced proteins. Tissue-enhanced proteins were produced by genes that were expressed at least four times higher than average mRNA levels in other tissues (17, 18), laying the basis of tissue subsets through single-sample gene set enrichment analysis using the R package GSVA (19).

### 2.6 Machine learning-based selection of biomarker

Based on significantly expressed molecules (absolute fold-change >1.5), the samples were split at 60% and 40% as training set and test set, respectively. These were then used to build machine learning diagnosis model. Receiver-operating characteristic (ROC) curve was performed to evaluate the diagnostic performance of the resulting model. A value of p < 0.05 was considered statistically significant.

### 2.7 Verification ELISA

The serum protein concentrations were measured using commercially available ELISA kits, following the manufacturer’s instructions.

### 2.8 Statistical analysis

Statistical analysis was carried out using GraphPad Prism 8.0 and R software. GraphPad Prism 9.5.1 was employed for the analysis of the quantitative results from the ELISA experiments. The student t test or ANOVA was utilized to determine significant differences between groups, with a p value of < 0.05 considered statistically significant.

## 3 Results

### 3.1 Plasma proteomic and metabolomic profile of OBI

**Figure 1:**
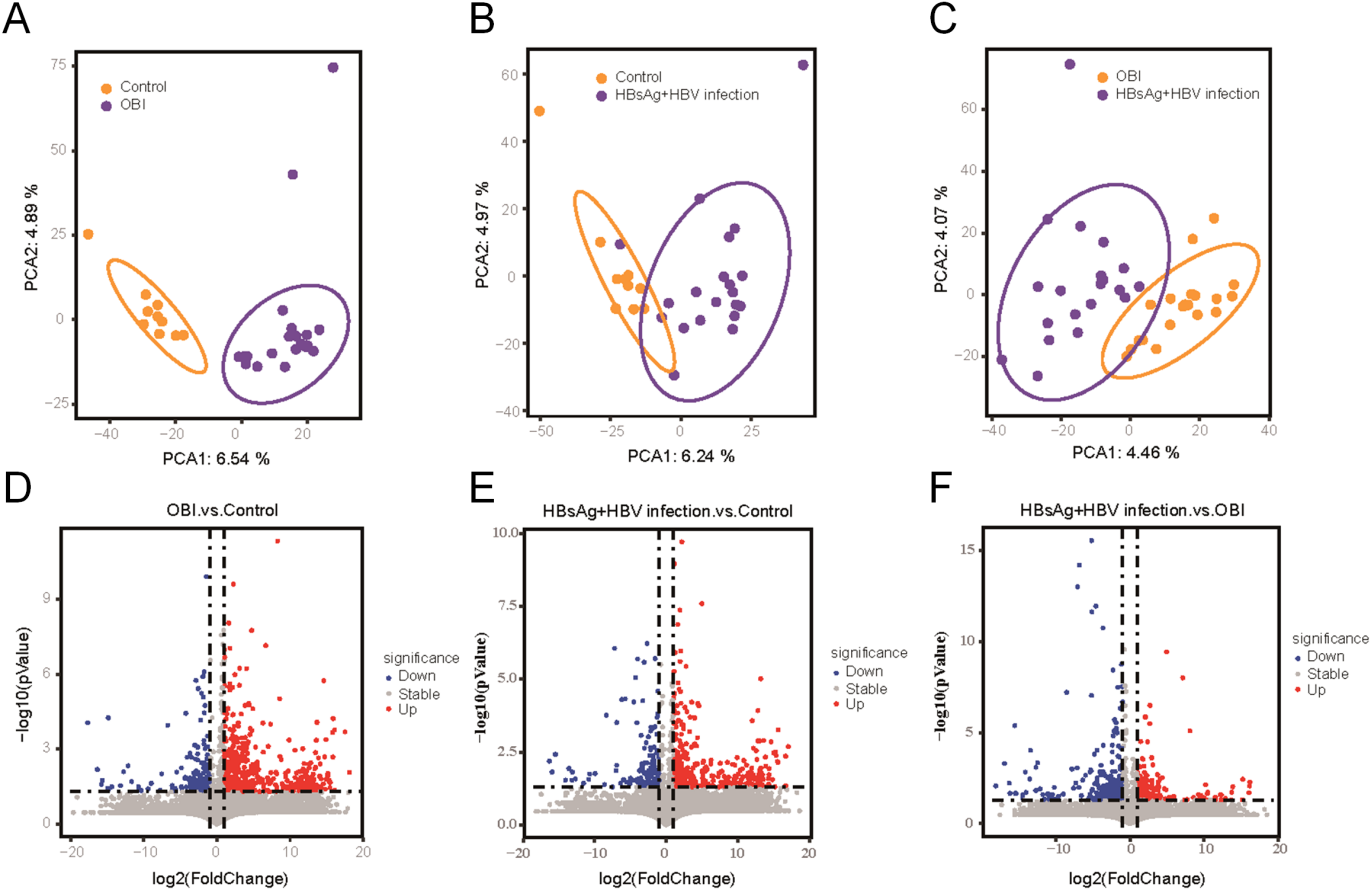
Differential proteins screened by proteomic analysis. **(A)** Principal components analysis (PCA) between OBI samples and control samples; **(B)** PCA between HBsAg-positive HBV infection samples and control samples; **(C)** PCA between OBI and HBsAg-positive HBV infection samples; **(D)** Volcano plot visualizing the differential protein expression in OBI samples compared to control samples; **(E)** The differential protein expression in HBsAg-positive HBV infection samples compared to control samples; **(F)** The differential protein expression in OBI samples compared to HBsAg-positive HBV infection samples.

The plasma proteomic and metabolomic landscape was investigated in 20 OBI patients, 20 HBsAg-positive HBV samples, and 10 healthy individuals with different demographic and clinical characteristics, including gender, age, HBV DNA level and ALT levels (**Table 1**). 7318 protein products were collected from the 50 individuals, with the number of proteins per sample ranging from 1800 to 2200. The identified number of coincident proteins among the three groups was 3947, where 349 proteins were specifically expressed in the control group, 710 proteins were separately expressed in the OBI group, and 737 proteins were specifically expressed in the HBsAg-positive group (**Supplementary Figure S1**). Principal components analysis (PCA) was utilized to assess if there were differences between the two groups. The OBI and control samples (**Figure 1A**), the HBsAg-positive and control samples (**Figure 1B**), as well as the OBI and HBsAg-positive samples (**Figure 1C**) were all distinguishable. The protein expression patterns between two groups were largely different, suggesting a notable variation in the protein expression profile. A volcano plot (**Figures 1D–F**) illustrates the proteins that were significantly increased and decreased between the indicated groups (|log2FoldChange|>1.5, p <0.05). A total of 1545 metabolites were identified during the process of metabolite identification, revealing noticeable variations in the expression of metabolites across various groups (**Supplementary Figures S2A–C**). Additionally, both upregulative and downregulative metabolites were distinguished by volcano plots (**Supplementary Figures S2D–F**). These findings disclose a significant variance in molecular expression among the three groups.

**Table 1:** Baseline information of enrolled patients and controls (revised).

Groups	Occult HBV infection (n=20)	HBsAg+ HBV infection (n=20)	Healthy control (n=10)	F/χ2	p value
Gender, n (%)^1^				2.188	0.335
Male	18 (90.0)	15 (75.0)	7 (70.0)		
Female	2 (10.0)	5 (25.0)	3 (30.0)		
Age, years^2^	46.65 ± 8.29	49.65 ± 14.82	40.70 ± 7.48		0.416
HBV DNA levels^3^	2.3 ± 0.16^1^	4.65 ± 1.91	N/A		<0.001
Log (IU/mL)					
ALT levels (U/L)^4^	18.39 ± 8.63	54.87 ± 57.46	23.70 ± 12.44		0.010

^1^Qualitative data were expressed in the form of number (%), and the differences among groups were compared using the chi-square test.

^2^Age values are presented as mean ± standard deviation, followed by analysis of the difference using a t test (between occult HBV infection and HBsAg+ HBV infection groups).

^3^5 patients HBV DNA level was positive but < 100 IU/mL, and recorded as below detectable limit. HBV DNA levels are presented as mean ± standard deviation, followed by analysis of the difference using a t test (between occult HBV infection and HBsAg+ HBV infection groups).

^4^ALT levels was represented as mean value ± std. deviation, followed by analysis of the difference using a t test (between occult HBV infection and HBsAg+ HBV infection groups).

p<0.05 was considered to be significant different.

### 3.2 Regulatory pathways and differentially expressed molecules

**Figure 2:**
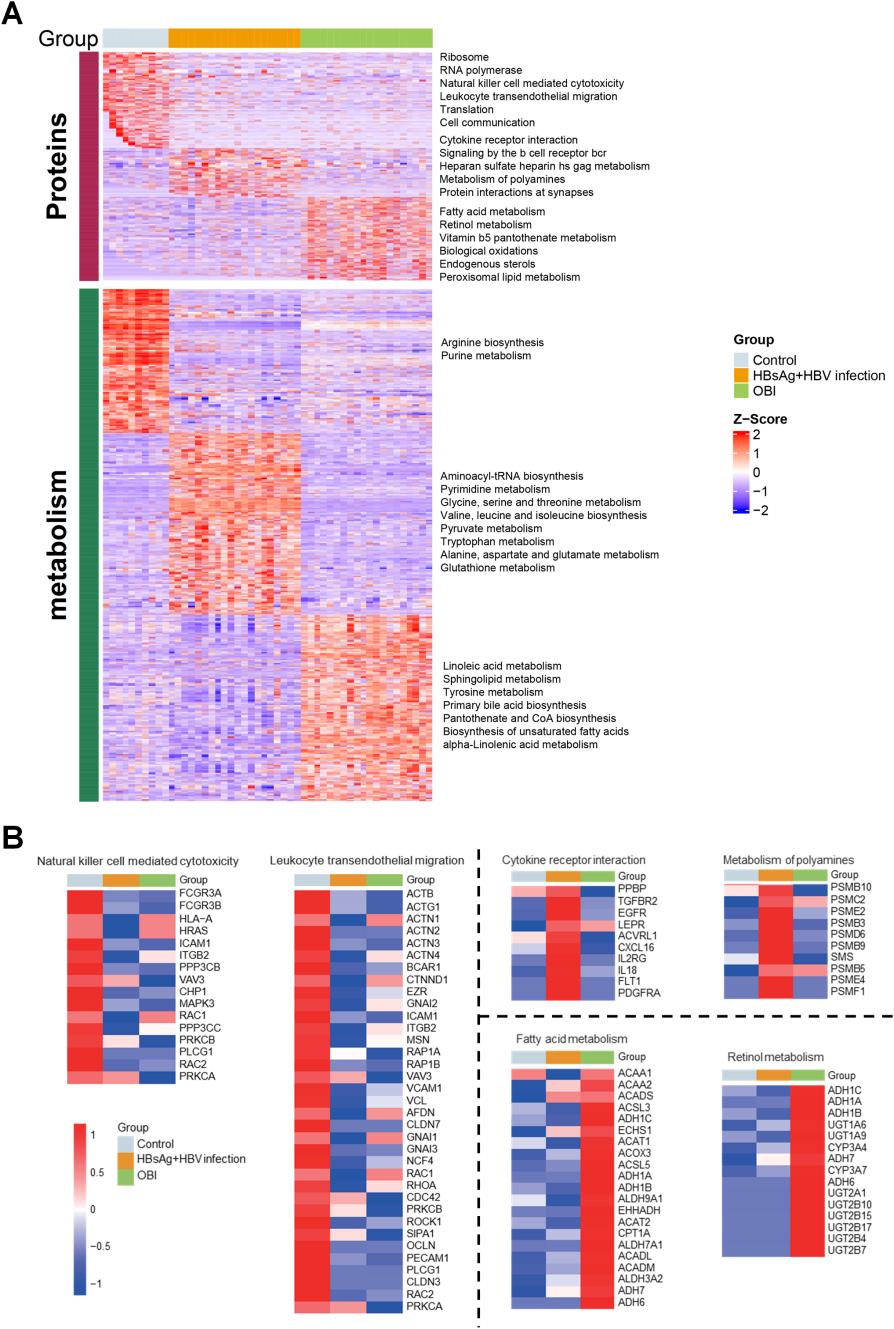
Regulatory pathways and differentially expressed molecules activated by multi-omic sequencing **(A)** Heatmap illustrating expression of differential molecules in the three groups, with each box in each row corresponding to one sample. The color intensity reflects the molecule’s expression level, with red representing high expression and blue denoting low expression. Pathways enriched for highly expressed protein molecules and metabolic molecules are identified on the right side. **(B)** Heatmap depicting the mean expression intensity of each protein in the characteristic pathways of the three groups, with different box colors representing specific protein expression. Proteins that show stronger expression in the group are highlighted in red, while proteins with lower expression in the group are displayed in blue.

Based on reactome pathway database, the pathways implicated by differential proteins and metabolites among groups were analyzed (**Figure 2A**). The enriched proteins or metabolites in healthy control group were associated with normal cellular functions, like ribosome, translation, and innate immunity. The dominant proteins or metabolites in HBsAg-positive HBV infection group were enriched in cellular inflammation, amino acid metabolism, and heparan sulfate metabolism. While in the OBI group, the dominant proteins or metabolites were associated with fatty acid metabolism, retinol metabolism, and oxidative stress. As shown in **Figure 2B**, we further identified highly expressed molecules in representative pathways of each group. In HBsAg-positive HBV infection, activation of cytokine receptor interaction and polyamines metabolism pathways leads to up-regulation of inflammatory factors (IL18, CXCL16), inflammatory-related receptors (EGFR, LEPR, IL2RG), and proteasome subunits. In occult HBV infection (OBI), genes related to alcohol dehydrogenase (ADH) and UDP-glycosyltransferase (UGT) superfamily were prominently expressed. These enzymes are responsible for alcohol and drug metabolism, which may contribute to the persistence of HBV infection in OBI.

### 3.3 Module-trait association revealed by WGCNA

**Figure 3:**
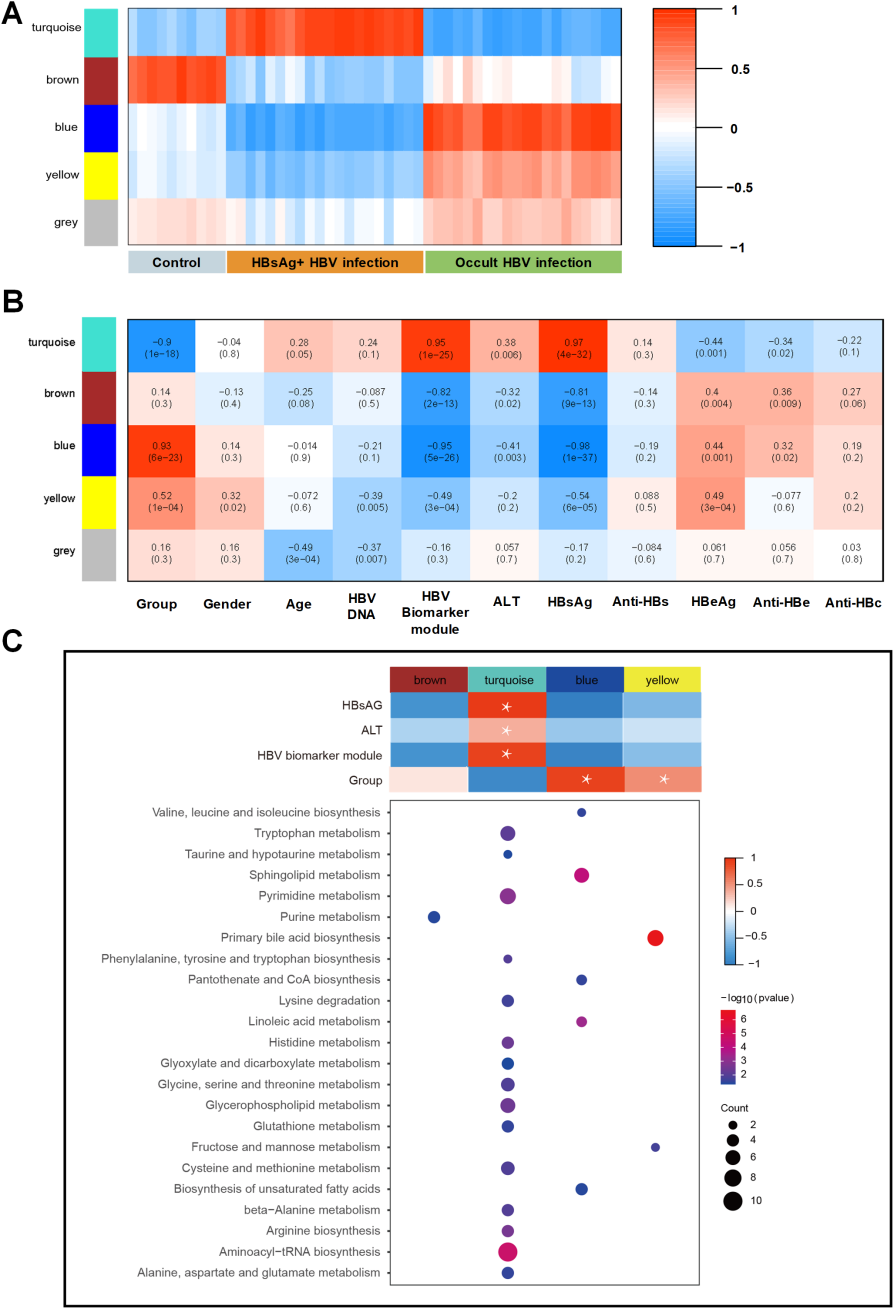
WGCNA identification of modules with highly correlated genes and their relationships with clinical variables. **(A)** The relationship between five distinct modules and different samples; **(B)** The relationship between modules and various clinical features; The correlation coefficient and p-value are presented in each box, with red denoting a positive correlation and blue denoting a negative correlation. A darker shade indicates a stronger correlation, with a correlation coefficient exceeding 0.25 indicating an interaction relationship. **(C)** Gene Ontology (GO) enrichment analysis was performed on four modules, and the enriched pathways are displayed on the left panel (Fisher-exact test, p<0.05).

To assess the relationship between distinct modules and different samples, WGCNA analysis was performed, and three groups were re-grouped as different colored modules (**Figure 3A**). Control group is represented by the brown module, HBsAg-positive group is represented by the turquoise module, and OBI group is represented by the blue, yellow, and grey modules. As shown in **Figures 3B, C**, the turquoise module, linked to the HBsAg-positive group, positively correlated with HBsAg, ALT, and HBV biomarker module. The activated pathways were mostly enriched in amino acid metabolism, including tryptophan and pyrimidine metabolism. The blue and yellow modules, linked to the OBI group, were enriched in pathways of primary bile acid biosynthesis and fatty acids. These results address that there are distinct molecular signatures and metabolic pathways associated with different stages of HBV infection, highlighting the complex interplay between viral infection and host metabolism in the progression of the disease.

### 3.4 Tissue damage caused by OBI

**Figure 4:**
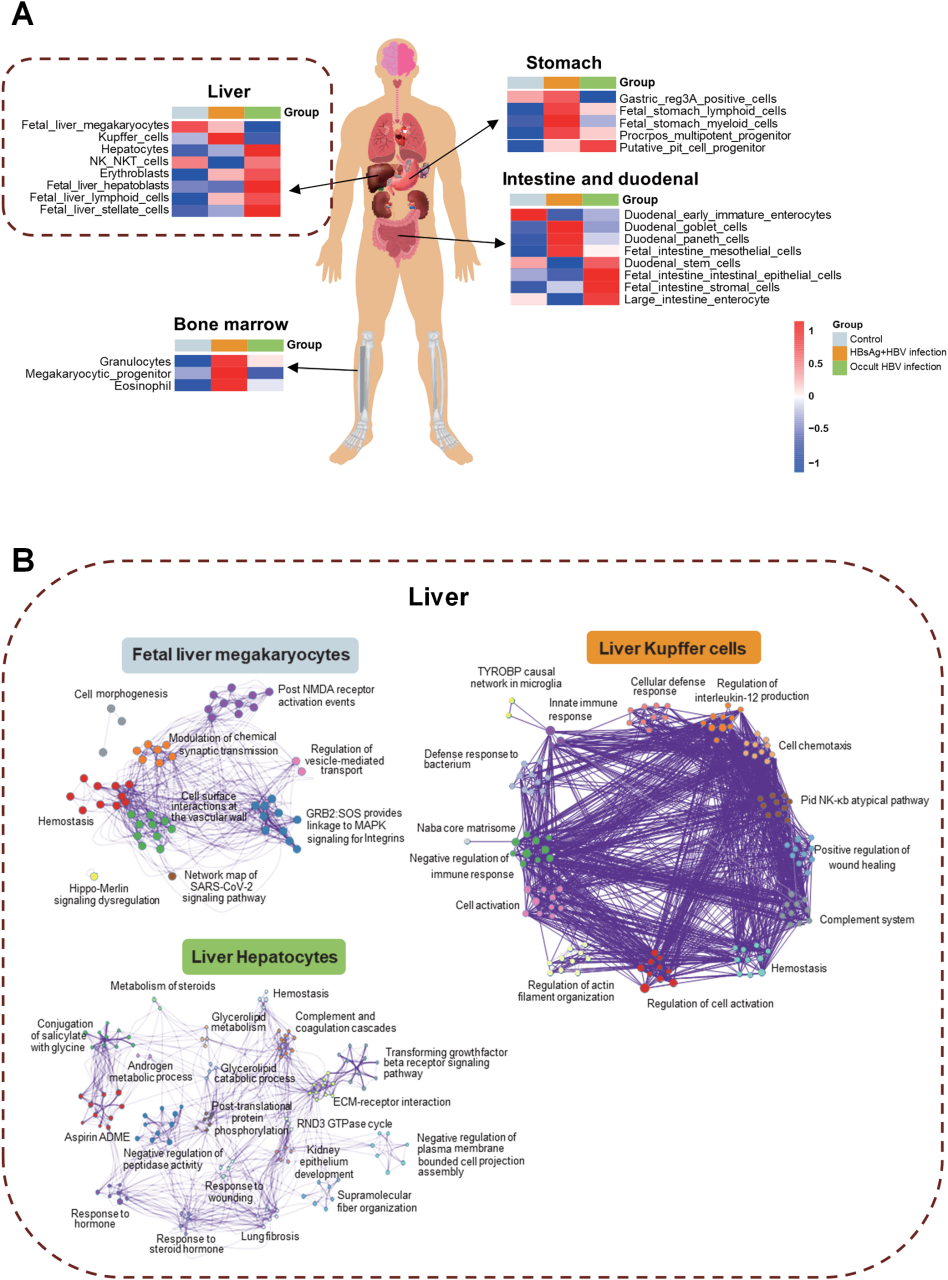
Tissue traceability analysis of OBI and HBsAg-positive HBV infection. **(A)** Tissue traceability analysis revealing the effects of HBsAg-positive HBV infection and occult HBV infection (OBI) on the liver, stomach, small intestine, duodenum, and bone marrow in patients; **(B)** The pathway interaction network diagram illustrating the interactions in different cell types of the liver triggered by various infection statuses.

HBV infection may have impact on various tissues of patients, including the liver, stomach, small intestine, duodenum, and bone marrow (**Figure 4A**). Identifying the different molecules and pathways that are expressed within specific tissues, we revealed that Kupffer cells in the liver, hematopoiesis-related functions in the bone marrow, lymphocytes, myeloid cells, and multifunctional progenitor cells in the stomach and goblet cells in the intestine were all affected in the HBsAg-positive infections. In the OBI group, liver parenchymal cells, NK NKT cells, erythroblasts, stem cells, intestinal stem cells, and intestinal epithelial cells were impacted. The pathway interaction network diagram (**Figure 4B**) illustrates the mutual regulatory interactions between the main pathways in fetal liver megakaryocytes, liver Kupffer cells, and hepatocytes. In hepatocytes of OBI patients, glycolipid, steroids metabolism, post-translational protein phosphorylation, and other inflammatory and metabolic pathways are interconnected. These findings corroborate that HBsAg-positive and negative HBV infection impact distinct intrahepatic cells and trigger separate pathways.

### 3.5 Immunopathological changes in OBI

**Figure 5:**
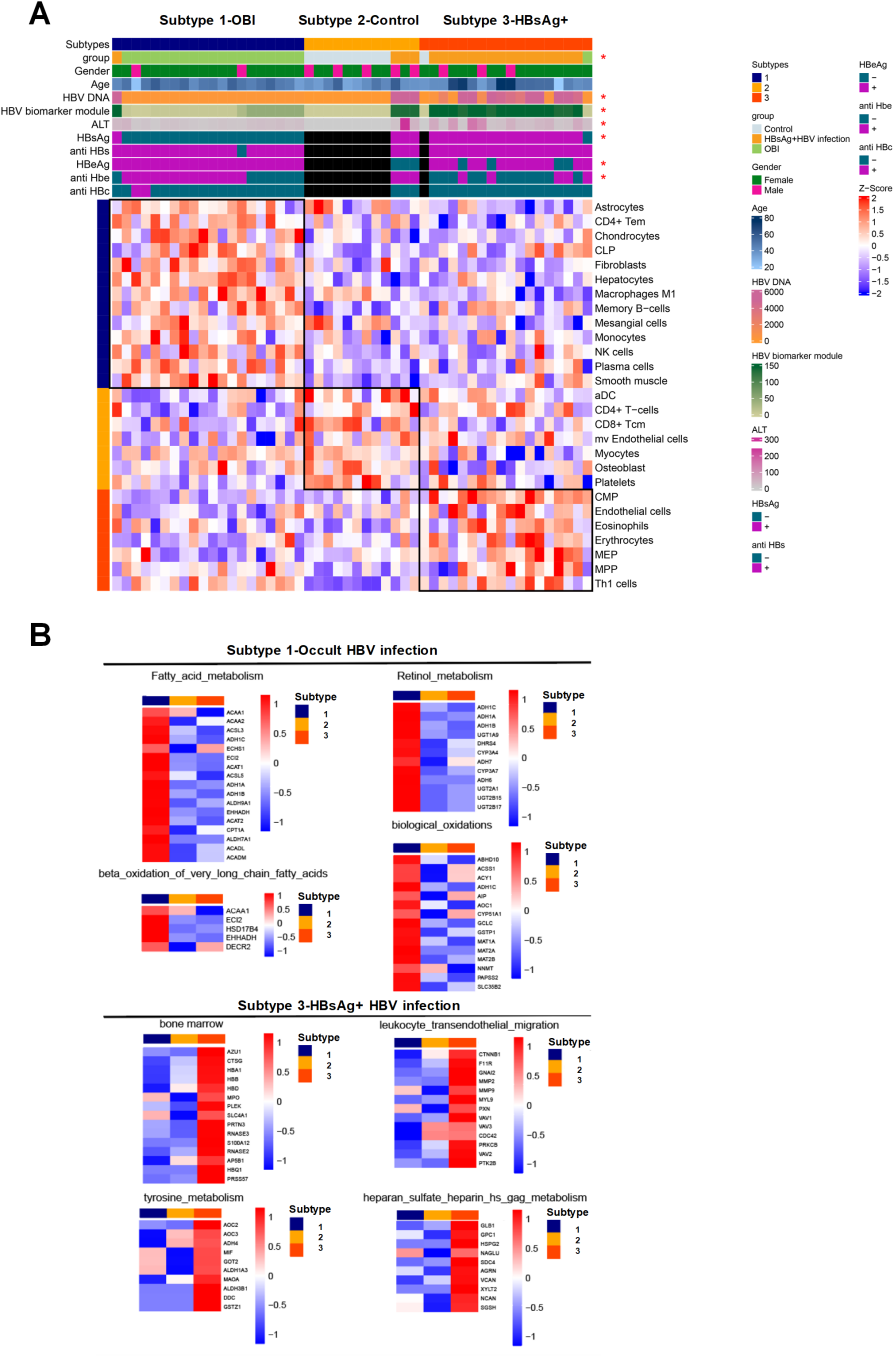
Characteristics of immune subtype with activated pathways and associated molecules. **(A)** Immunophenotyping categorizing the patients into three subtypes. Subtype 1 consisting mainly of OBI patients, subtype 2 mainly including control samples, and subtype 3 primarily comprising HBsAg-positive HBV infected patients. The heatmap indicating clinical features significantly correlated with immunophenotyping (p<0.05) with an asterisk. **(B)** Heatmap displaying the pathways that exhibit significant positive correlations with subtypes 1 and 3, along with the expression levels of specific molecules within those pathways. The intensity of color reflects the expression level of each molecule on the right side of the heatmap. Red signifies higher expression levels, while blue indicates lower expression levels. The collective molecular expression serves as a representation of pathway alterations.

The patients were categorized into three types based on immunophenotyping (**Figure 5A**), each displaying distinct immune characteristics. Type 1 consisted mainly of OBI patients with predominant immune cells being CD4 Tem and memory B cells. Type 2 was mainly comprised of normal samples with immune cells primarily consisting of dendritic cells and monocytes. Type 3 consisted mainly of HBsAg-positive HBV infected patients, with immune cells mainly being red blood cells and bone marrow-related progenitor cells. The overall molecular expression of pathway changes revealed that in type 1 (OBI patients), there was a significant up-regulation of pathways such as fatty acid metabolism and biological oxidation. In type 3 (HBsAg-positive HBV patients), pathways such as bone marrow and amino acid metabolism and leukocyte transepithelial migration were significantly up-regulated (**Figure 5B**). These results show that OBI has a distinct immunophenotype and molecular pathway compared to both normal samples and HBsAg-positive HBV patients.

### 3.6 Machine learning-based selection of OBI plasma biomarkers

**Figure 6:**
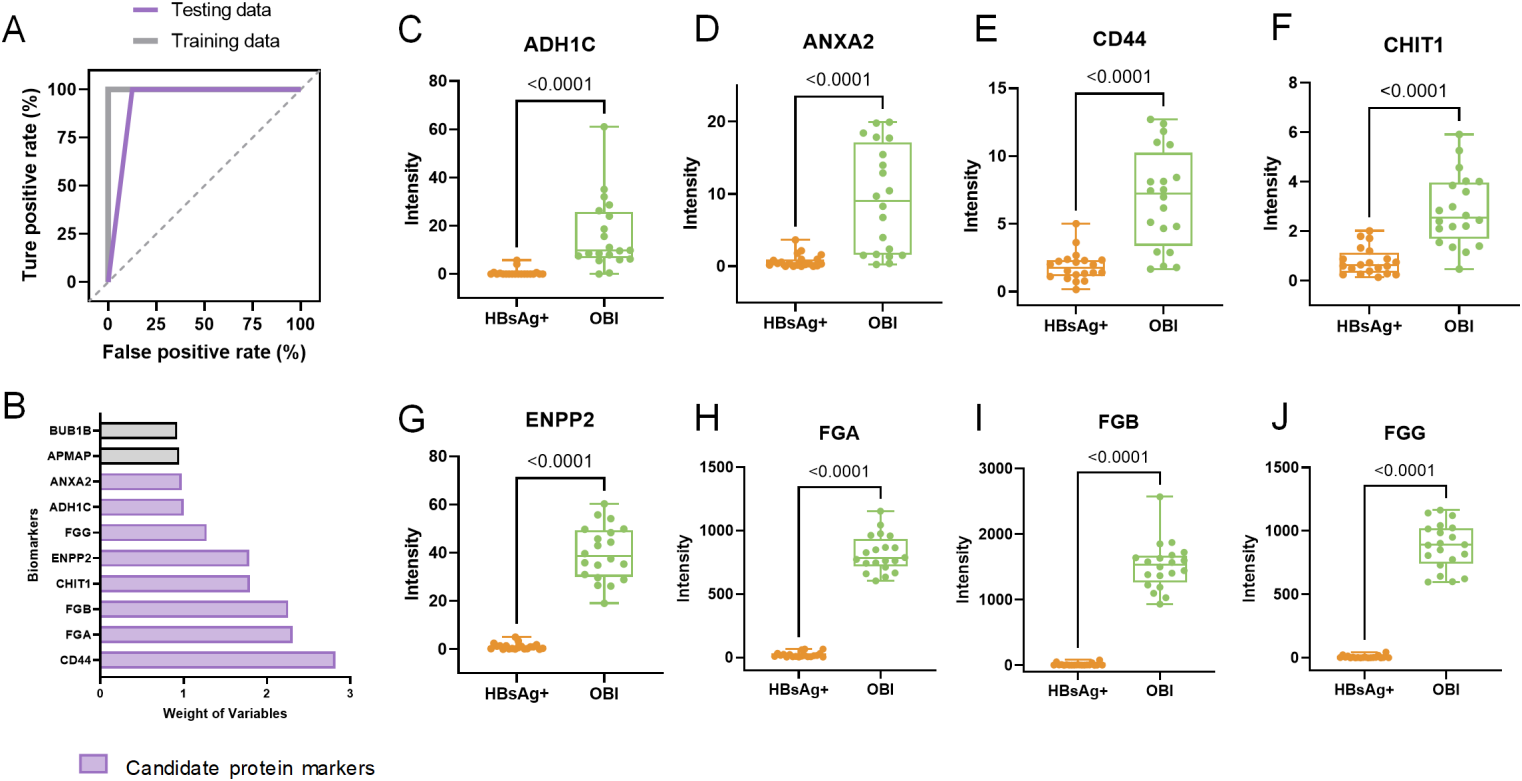
Machine learning-based selection of protein biomarkers and expression of candidate markers. **(A)** Receiver operating characteristic (ROC) analysis evaluating the effectiveness of diagnostic model; **(B)** The bar graph displays the selected protein markers, which were chosen using various methods and given comprehensive weights. The weight value indicates the markers’ contribution to distinguishing between the two groups. Black bars represent molecules that did not meet the screening criteria, while purple bars indicate potential biomarker candidates. **(C-J)** The expression levels of eight selected protein markers analyzing with liquid chromatography-tandem mass spectrometry (LC-MS).

When comparing HBV infected samples with healthy controls, the AUC value for the training set was 1 (p<0.001), indicating perfect discrimination, while the AUC value for the testing data was 0.859 (**Supplementary Figure S3**). Five markers which were overexpressed in HBV infection group (containing HBsAg-positive HBV infection and OBI) were selected for distinguishing HBV samples from control. The cohort was split into a training dataset and a test dataset based on the expression levels of protein markers that differed significantly between individuals with OBI and HBsAg-positive HBV infection. A diagnostic model utilizing machine learning was developed using these markers to differentiate between OBI and HBsAg-positive HBV infection samples. The AUC values for the training and testing sets were 1 and 0.9375, respectively (**Figure 6A**). By combining multiple feature selection methods, markers were scored with comprehensive weights to determine their contribution to distinguishing the two comparison groups. Eight markers were selected for distinguishing OBI and HBsAg-positive group (**Figure 6B**). Their expression levels are shown in **Figures 6C–J**, and they were all found to be overexpressed in the OBI group (p<0.001).

**Figure 7:**
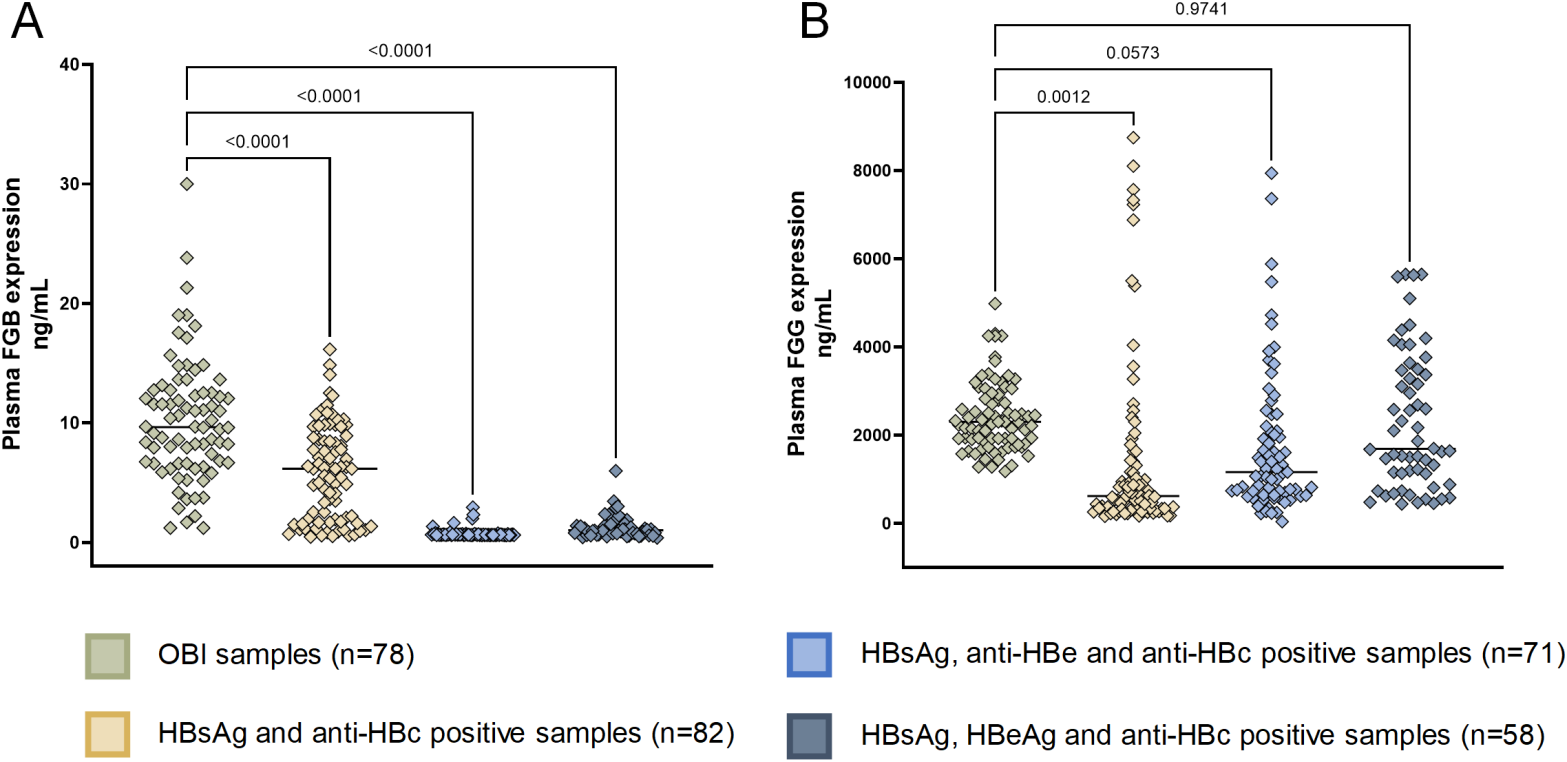
ELISA of FGB and FGG in validation cohort. **(A)** Human Fibrinogen beta chain **(FGB)** expression in patients with OBI and HBsAg-positive HBV infection (detected using the ELISA kit CSB-E09611h, CUSABIO, China with a detection range of 0.45 ng/ml-30 ng/ml, using the sandwich method); **(B)** Human Fibrinogen Gamma Chain (FGG) expression in patients with OBI and HBsAg-positive HBV infection (detected using the ELISA Kit CSB-E13319h, CUSABIO, China with a detection range of 125 ng/mL-8000 ng/mL, using the competitive method).

We then validated the expression of eight OBI-specific markers in discovery cohort with ELISA. The expression of eight markers can be seen in **Supplementary Figure S4**, we finally selected FGB and FGG, which yielded consistent results in ELISA and LC-MS, for further validation. FGB was overexpressed in OBI samples compared to other three HBsAg-positive HBV infection status (**Figure 7**). While FGG was only overexpressed in OBI samples compared to HBsAg and anti-HBc positive samples (**Figure 7B**, p=0.0012).

In the selection of metabolites, the AUC value for distinguishing HBsAg-positive HBV infection and OBI group was 1 (p<0.001) for the training data and 0.9297 (p=0.039) for the testing data. Twenty metabolome biomarkers were selected, with eight markers being overexpressed in the OBI group and twelve markers being down-regulated (**Supplementary Figures S5A–C**). In this study, various proteins and metabolites were discovered as potential markers of OBI and HBV infection.

## 4 Discussion

**Figure 8:**
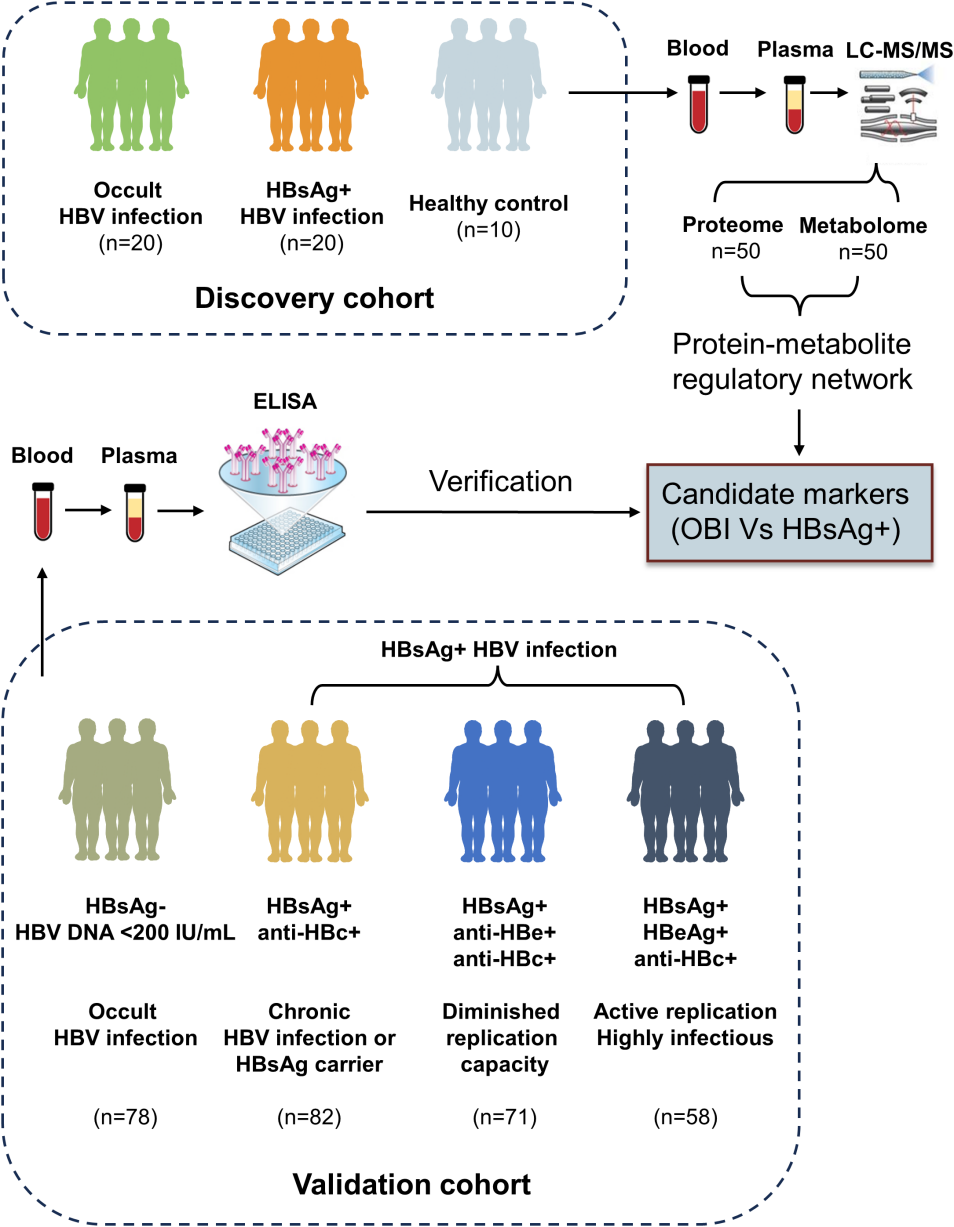
We examined 20 samples of OBI, 20 samples with HBsAg-positive HBV infection, and 10 healthy controls. We analyzed plasma samples for proteome and metabolome to investigate the protein-metabolite regulatory network. We identified several potential markers that could distinguish between OBI and HBsAg-positive samples. These markers were further validated using ELISA in a larger cohort of 78 OBI samples, 82 HBsAg-positive samples with anti-HBc positivity, 71 HBsAg-positive samples with anti-HBe and anti-HBc positivity, and 58 HBsAg-positive samples with HBeAg positivity and anti-HBc positivity. OBI, Occult HBV infection; LC-MS/MS, Liquid chromatography-tandem mass spectrometry; ELISA, Enzyme linked immunosorbent assay.

This study describes the immune microenvironment in the peripheral circulation of HBsAg-positive HBV patients and OBI patients using multi-omics approach. It assessed the pathological and physiological characteristics, immune levels, and metabolic status of these patients (**Figure 8**). Additionally, a diagnostic model constructed by machine learning demonstrated strong classification performance and potential diagnostic value. These findings offer a new non-invasive approach to identifying OBI and HBsAg-positive HBV infections.

In this study, we gained consistent results with previous studies. We found the higher ALT levels and older age observed in HBsAg-positive patients indicate a longer duration of infection and poorer liver function. The ALT levels were higher in patients with HBV-related hepatitis, which was proceeded by the peak HBV DNA level (20). In HBsAg-positive patients, their HBV DNA was higher than OBI patients (**Table 1**, p<0.001). Tissue tracing in the liver revealed a relationship between Kupffer cells and HBsAg-positive HBV infection, as well as between OBI and hepatocytes. It was reported that the functionality of Kupffer cells is altered by the HBV, aiding in the establishment and potential maintenance of infection. HBV hinders the production of the antiviral cytokine IL-1β, while simultaneously enhancing the production of IL-10 in the microenvironment (21). As the relationship between OBI and hepatocytes, although the viral load in OBI may not be as high as in HBsAg-positive infection, the long-term stable presence of covalently closed circular DNA in the nucleus of infected hepatocytes is what underlies the development of OBI (1, 22). The liver’s specific cell types are influenced by different states of HBV and viral proteins, which in turn activate relevant pathways.

Immune profiling indicated the presence of CD4 Tem and memory B cells in OBI. These cells have the ability to rapidly respond to infection reactivation by secreting cytokines and producing antibodies, potentially inhibiting viral protein secretion and binding to viral proteins. There is currently no other research indicating a connection between CD4 Tem and OBI, while a multi-omics study on HCC revealed a notable rise in the percentage of CD4 Tem in patients with unfavorable outcomes (23). However, memory B cells was reported showing signs of activation in both the blood and liver of patients with high HBsAg levels (24). While memory B cells were abundant in OBI patients with undetectable HBsAg in this study, the differences between the two studies may be attributed to the sampling methods and sample sizes. Also, the previous studies have focused on activation markers of memory B cells, such as the early activation marker CD69, while our study solely concentrated on the enrichment of classical markers of relevant cells. Moving forward, we also aim to investigate specific subsets within memory B cells and explore the molecular differences in their expression. On the other hand, the presence of erythroid progenitor cells in HBsAg-positive patients indicates that HBV infection could potentially impact the differentiation of erythroid and myeloid lineages. Studies have shown that HBV can inhibit hematopoiesis and result in severe bone marrow failure, as evidenced by cases of anemia, macrocytosis, and significant thrombocytopenia (25, 26). However, more clinical cases and further exploration of the underlying mechanisms are needed to fully understand the impact of HBV on hematopoiesis.

After constructing a diagnostic model, we found that the ROC curve showed a significant classification performance for the model. Both FGB and FGG were found to be overexpressed in OBI patients compared to those with HBsAg-positive HBV infection, making them potential biomarkers for distinguishing the two conditions. FGB and FGG have the ability to polymerize and produce an insoluble fibrin matrix known as fibrinogen. It has been reported that FGB and FGG are higher in the plasma exosomes of malignant pulmonary nodules, and have ability to distinguish benign from malignant pulmonary nodules (27). They are also associated with infection and can enhance anti-bacterial immune responses, being the target genes for STAT3 and IL-6 pathways (28). Furthermore, they have been recognized in several types of cancer (29, 30). They can be biomarkers and promoting factors of cancer, by enhancing the proliferation, invasion, metastasis and cancer-associated fibroblast recruitment of cancer cells (31). In HCC, FGB functions in portal vein tumor thrombus formation (32), FGG activating epithelial to mesenchymal transition to promote migration and invasion in hepatocellular carcinoma cells (33). The elevated expression of FGB and FGG have also been observed in another HCC-related research (34, 35). In the present study, FGB and FGG were overexpressed in the plasma of OBI patients. OBI is a risk factor for HCC (7), and we speculate that patients with OBI are also at risk of developing hepatocellular carcinoma. FGB and FGG may contribute to the occurrence and progression of OBI. Their high expression in the plasma circulation also makes them easy to detect. Thus, the detection of these two markers in OBI patients may help monitor the progression of OBI.

In the validation of larger sample cohorts, we separated HBsAg-positive samples into three groups: 1) HBsAg, HBeAg and anti-HBc positive samples; 2) HBsAg, anti-HBe and anti-HBc positive samples; 3) HBsAg and anti-HBc positive samples. These three groups represented different stages of hepatitis B infection. HBsAg, HBeAg and anti-HBc positive samples have higher ALT levels (**Supplementary Table S1**), showing the most severe liver damage. With the course of the disease, HBeAg goes through seroconversion, firstly becomes anti-HBe and then disappears, which corresponds with the serological features of the other two groups. With the progression of the disease to the occurrence of OBI, the FGB shows a gradual increase, which may be related to the chronicity of liver disease and the risk of HCC.

There are also some disadvantages in this study. First, eight candidate proteins were identified but only three proteins showed consistent and significant differential expression when validated by ELISA. This may be due to the different detection principles, modes and sensitivities of the two methods. Proteomics is advantageous for screening many differentially expressed proteins, however, some of them cannot be verified by simple and economical protein detection methods. For the final application and subsequent research and development, we focused on FGB and FGG, which yielded consistent quantitative results in both ELISA and LC-MS. Second, we did not include the molecular characteristic of HBV, as the mutation on HBV genome may be also associated with the abnormal expression of FGB and FGG. Third, the number of samples included was limited, though more data collection is planned in the future. Lastly, some markers of metabolomics have not been studied or explored. In the future, we hope to reveal their functions and upstream regulators.

In conclusion, our work describes the multi-omic molecular characterization of OBI, constructs a machine learning-based diagnostic model with impressive accuracy and identifies two potential OBI biomarkers. It highlights a non-invasive strategy for characterizing OBI patients who may not be clinically discriminative, and may help improve treatment course or clinical outcomes in patients with special HBV infection status.

## Data Availability

The datasets generated in this research are accessible in the following databases: the raw mass spectrometry data is available on iProX (https://www.iprox.cn//page/project.html?id=IPX0009413000), with the project ID IPX0009413000. The raw data for metabolomics can be accessed on MetaboLights (https://www.ebi.ac.uk/metabolights/MTBLS11406) under project ID MTBLS11406. Both datasets are publicly available.
