# Supplementary material for: Multi-omic molecular characterization and diagnostic biomarkers for occult hepatitis B infection and HBsAg-positive hepatitis B infection
Source: Front Endocrinol (Lausanne). 2024 Nov 12;15:1409079. doi: 10.3389/fendo.2024.1409079 (PMC11588476; doi:10.3389/fendo.2024.1409079)
Supplement: Supplementary file 1 [file DataSheet1.docx]

Supplementary Material

## Supplementary table 1. Baseline information of the validation cohort (revised)

| Groups | Occult HBV infection (n=78) | HBsAg and anti-HBc positive (n=82) | HBsAg, anti-HBe and anti-HBc positive (n=71) | HBsAg, HBeAg and anti-HBc positive (n=58) | F/χ2 | p value |
| --- | --- | --- | --- | --- | --- | --- |
| Sex, n (%) |  |  |  |  | 7.78 | 0.051 |
| Male | 45 (57.7) | 62 (75.6) | 48 (67.6) | 33 (56.9) |  |  |
| Female | 33 (42.3) | 20 (24.4) | 23 (32.4) | 25 (43.1) |  |  |
| Age, years^3^ | 42.26 ± 7.89 | 38.18 ± 9.64 | 51.93 ± 13.93 | 38.79 ± 12.46 | 23.56 | <0.001 |
| HBV DNA levels Log(IU/mL) | 2.23 ± 0.12^1^ | 3.50 ± 0.96^2^ | 3.88 ± 1.13 | 5.81 ± 2.35 | 67.88 | <0.001 |
| ALT levels (U/L)^3^ | 21.81 ± 9.22 | 19.27 ± 8.59 | 50.65 ± 72.43 | 113.4 ± 189.4 | 14.47 | <0.001 |

^1^ 19 patients HBV DNA level was positive but below 100 IU/mL, and recorded as below detectable limit

^2^ 23 patients HBV DNA level was positive but below 100 IU/mL, and recorded as below detectable limit

^3^ Age, ALT and HBV DNA levels was represented as mean value ± std. deviation, followed by analysis of the difference using one-way analysis of variance (among four groups).

## Supplementary Figures


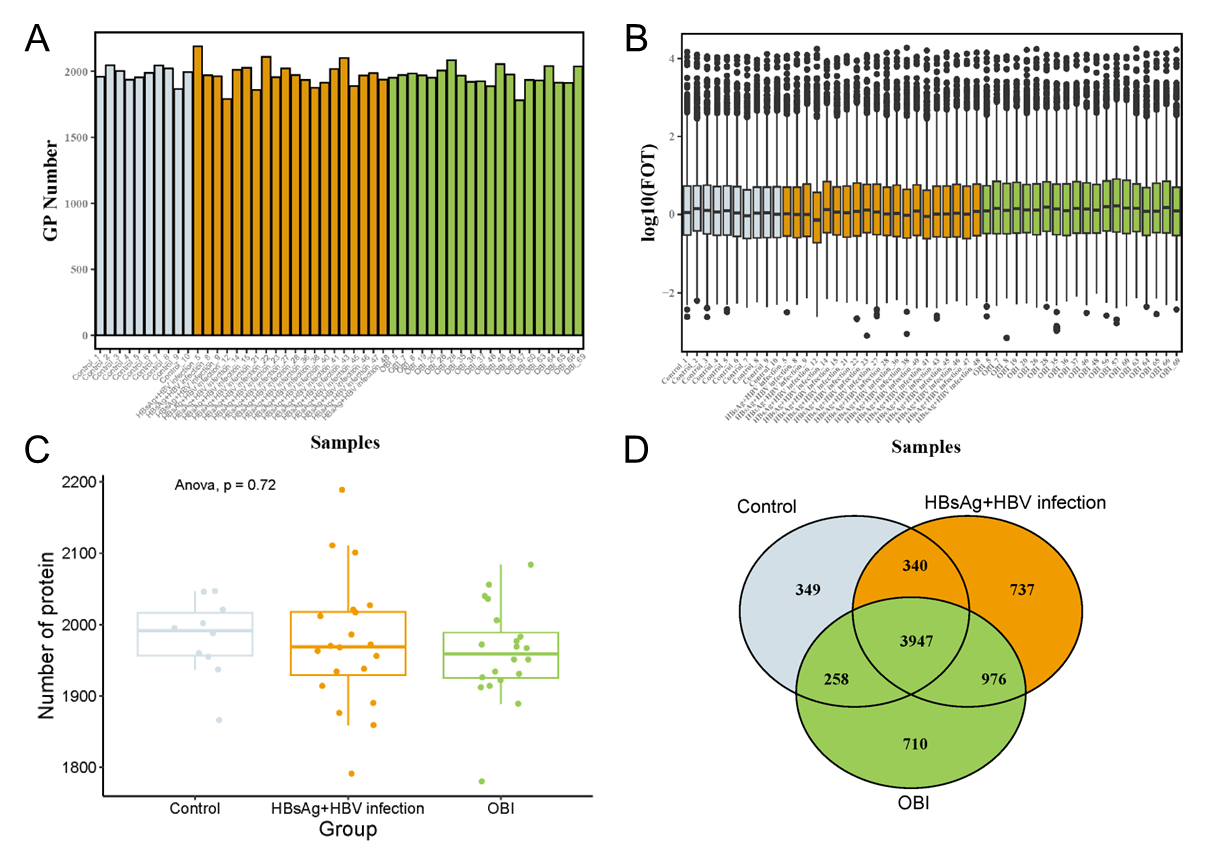


**Supplementary Figure 1. Identification number and abundance analysis of the proteome results. A.** Bar chart showing the number of proteins identified in each individual sample. **B.** Box plot indicating the protein abundance of each sample. **C.** Comparison of the numbers of identified proteins in different groups. **D.** Venn plot illustrating the overlap of proteins among the three groups.


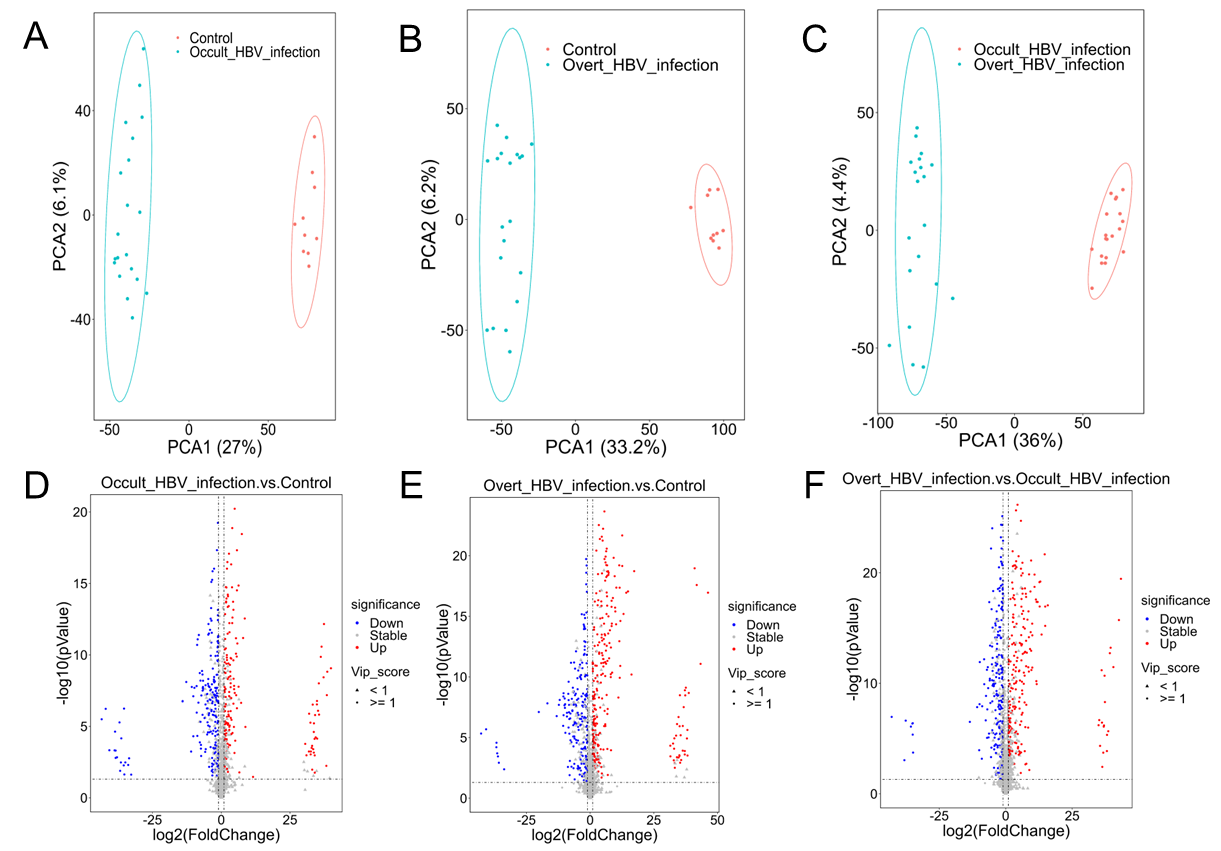


**Supplementary Figure 2. The expression and differential metabolite molecules in three groups. A-C.** PCA plots identifying the differences in metabolite molecules between indicated groups. **~~B~~D-F.** Volcano plots visualizing the differential metabolite expression between indicated groups.


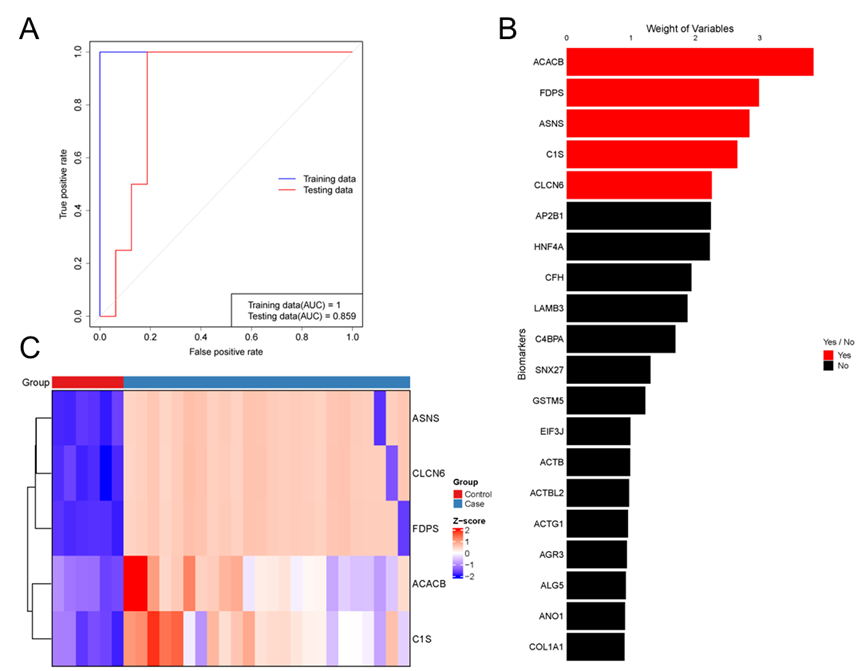


**Supplementary Figure 3. Machine learning-based selection of candidate protein markers distinguishing HBV infection and healthy control. A.** ROC analysis evaluating the diagnostic model’s performance. The cohort was split into a training set and a test set based on the levels of protein markers that showed significant differences between individuals with HBV infection (including OBI and HBsAg-positive HBV infection) and healthy controls. A diagnostic model utilizing machine learning was created using these markers to distinguish between HBV infection and controls. **B.** Bar graph displaying the chosen protein markers. The weight value indicating the importance of the markers in distinguishing between the two groups. Red bars indicating potential biomarker candidates. **C.** Heatmap showing the expression of five selected protein markers.


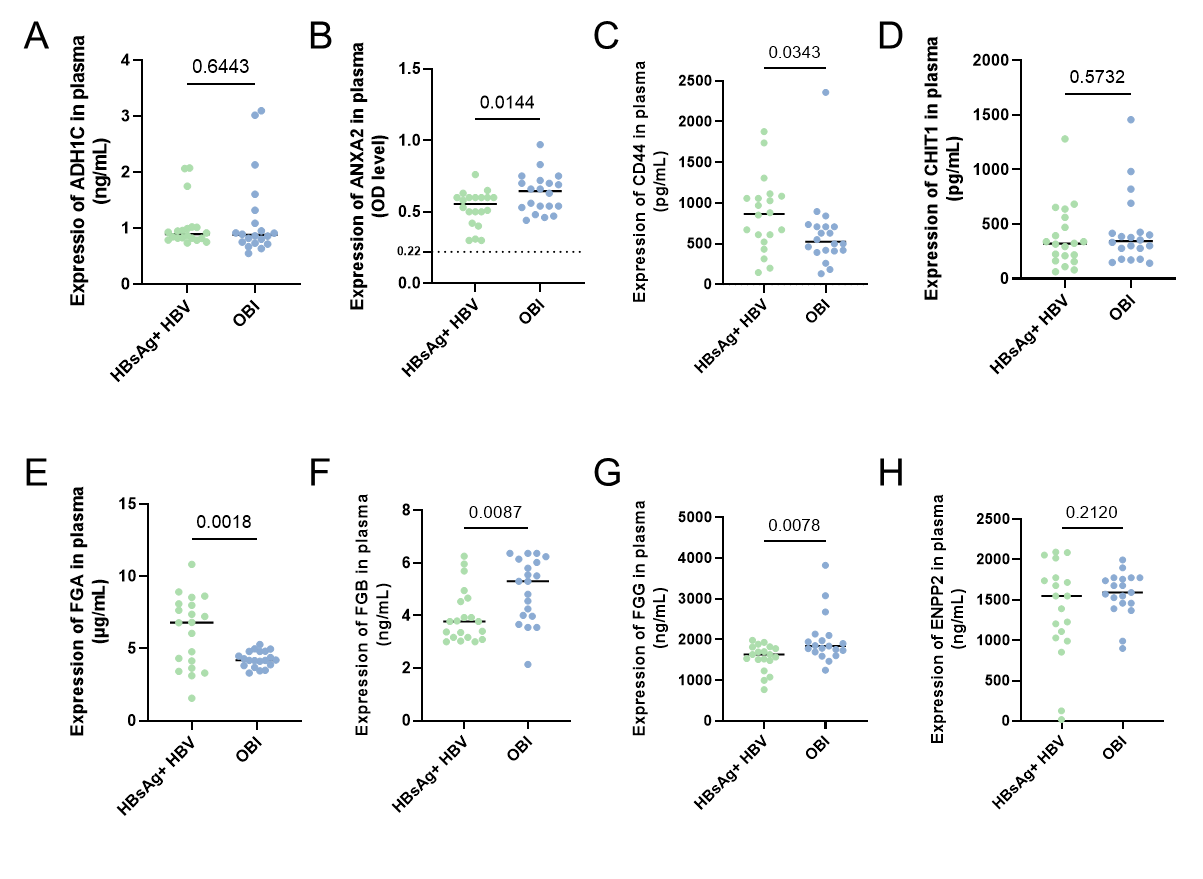


**Supplementary Figure 4. ELISA validation for selected protein markers.** ELISA validating expression of eight protein markers in the discovery cohort. The serum protein concentrations were measured using commercially available ELISA kits. **A.** The expression of ADH1C (detection range: 0.312-20 ng/mL, E11343h, EIAab, China); **B.** Qualitative detection of ANXA2 (CSB-E17660h, CUSABIO, China); **C.** The expression of CD44 (78-5000 pg/mL, CSB-E11846h, CUSABIO, China); **D.** The expression of CHIT1 (31.25-2000 pg/mL, CSB-E17930h, CUSABIO, China); **E.** The expression of FGA (0.94-60 μg/mL, CSB-EL008607HU, CUSABIO, China); **F.** The expression of FGB (0.45-30 ng/mL, CSB-E09611h, CUSABIO, China); **G.** The expression of FGG (125-8000 ng/mL, CSB-E13319h, CUSABIO, China); **H.** The expression of ENPP2 (25-2000 μg/mL, CSB-EL007680HU, CUSABIO, China).


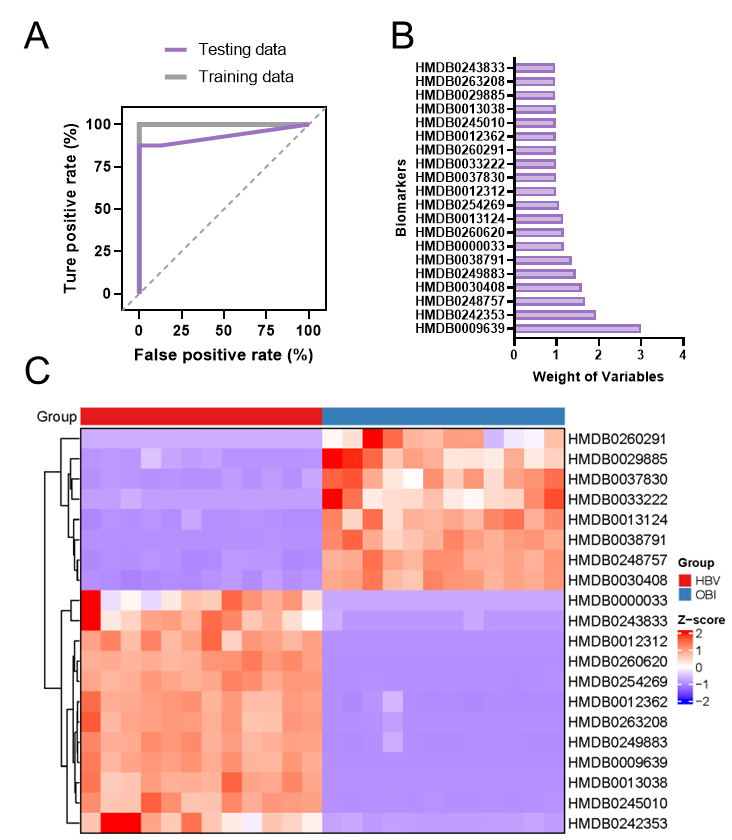


**Supplementary Figure 5. Machine learning-based selection of candidate metabolite biomarkers. A.** ROC analysis evaluating the performance of diagnostic model. The cohort was divided into a training set and a test set based on significant differences in metabolite markers between individuals with occult hepatitis B infection (OBI) and HBsAg-positive HBV infection. The diagnostic model using machine learning was developed with these markers to differentiate between the two groups. **B.** Bar graph displaying the selected metabolite markers, with weight values indicating their importance in distinguishing between the groups. Purple bars representing potential biomarker candidates. **C.** Heatmap visualizing the expression of 20 chosen metabolite markers, with 8 markers showing higher expression and 12 showing lower expression in the OBI group.
